# Tumor-Targeting Anti-CD20 Antibodies Mediate *In Vitro* Expansion of Memory Natural Killer Cells: Impact of CD16 Affinity Ligation Conditions and *In Vivo* Priming

**DOI:** 10.3389/fimmu.2018.01031

**Published:** 2018-05-11

**Authors:** Cristina Capuano, Simone Battella, Chiara Pighi, Lavinia Franchitti, Ombretta Turriziani, Stefania Morrone, Angela Santoni, Ricciarda Galandrini, Gabriella Palmieri

**Affiliations:** ^1^Department of Experimental Medicine, Sapienza University of Rome, Rome, Italy; ^2^Department of Molecular Medicine, Sapienza University of Rome, Rome, Italy; ^3^Laboratorio Pasteur Italia Fondazione Cenci Bolognetti, Sapienza University of Rome, Rome, Italy; ^4^IRCCS Neuromed, Pozzilli, Italy

**Keywords:** memory NK cells, cancer immunotherapy, therapeutic anti-CD20 mAb, CD16, *in vitro* expansion

## Abstract

Natural killer (NK) cells represent a pivotal player of innate anti-tumor immune responses. The impact of environmental factors in shaping the representativity of different NK cell subsets is increasingly appreciated. Human cytomegalovirus (HCMV) infection profoundly affects NK cell compartment, as documented by the presence of a CD94/NKG2C^+^FcεRIγ^-^ long-lived “memory” NK cell subset, endowed with enhanced CD16-dependent functional capabilities, in a fraction of HCMV-seropositive subjects. However, the requirements for memory NK cell pool establishment/maintenance and activation have not been fully characterized yet. Here, we describe the capability of anti-CD20 tumor-targeting therapeutic monoclonal antibodies (mAbs) to drive the selective *in vitro* expansion of memory NK cells and we show the impact of donor’ HCMV serostatus and CD16 affinity ligation conditions on this event. *In vitro* expanded memory NK cells maintain the phenotypic and functional signature of their freshly isolated counterpart; furthermore, our data demonstrate that CD16 affinity ligation conditions differently affect memory NK cell proliferation and functional activation, as rituximab-mediated low-affinity ligation represents a superior proliferative stimulus, while high-affinity aggregation mediated by glycoengineered obinutuzumab results in improved multifunctional responses. Our work also expands the molecular and functional characterization of memory NK cells, and investigates the possible impact of CD16 functional allelic variants on their *in vivo* and *in vitro* expansions. These results reveal new insights in Ab-driven memory NK cell responses in a therapeutic setting and may ultimately inspire new NK cell-based intervention strategies against cancer, in which the enhanced responsiveness to mAb-bound target could significantly impact therapeutic efficacy.

## Introduction

The perspective of natural killer (NK) cells as exquisitely innate effectors is challenged by the recent appreciation that long-lasting NK cell populations with enhanced effector functions may arise in response to environmental factors, named adaptive or memory NK cells (1–3).

*In vivo*, memory NK cells have been identified in human cytomegalovirus (HCMV)-seropositive subjects, mainly on the basis of high expression levels of CD94/NKG2C activating receptor and CD57 terminal differentiation marker (4, 5). Recent studies have identified an immune-receptor tyrosine-based activation motif (ITAM)-bearing FcεRIγ adaptor protein-deficient (FcεRIγ^−^) NK cell subset in HCMV-seropositive individuals, endowed with a specific epigenetic signature and mostly overlapping with the CD94/NKG2C^high^ population (6–10). FcεRIγ chain deficiency thus represents the hallmark of memory NK cell population, together with variable loss of Syk and EAT2 signaling intermediates, low amount of the pro-myelocytic leukemia zinc finger (PLZF) transcription factor, and a distinctive genome-wide methylation profile similar to that of memory effector T cells (8, 9). As a consequence of FcεRIγ deficiency, memory NK cells exhibit lower surface levels of FcεRIγ-dependent NKp46 and NKp30 activating receptors, with respect to FcεRIγ^+^ conventional NK cells (1, 6).

Memory NK cells can constitute up to 70% of the total NK cell population in HCMV-seropositive individuals. The mechanistic basis of their selective expansion is incompletely understood. It has been proposed that primary HCMV infection drives the priming and proliferation of memory NK cells in a NKG2C-dependent manner (1–3, 11, 12). Such expansion may be maintained by a variety of different viral super-infections. In particular, an expanded population of memory NK cells was detected in EBV-, HBV-, HCV-, and HIV-seropositive individuals, only when patients were also seropositive for HCMV (13, 14). It is therefore conceivable that Ab-mediated immune responses may drive the proliferation and maintenance of an already existing pool of memory NK cells, in some viral disease settings. Seminal *in vitro* studies offered a mechanistic explanation for the role of virus specific Abs in sustaining memory NK cell expansion, establishing a pivotal role for CD16 binding to Ab-opsonized infected cells (8, 9).

CD16, the low-affinity Fc receptor for IgG, or FcγRIIIa, represents a prototype NK activating receptor; its engagement by IgG-opsonized targets is sufficient to trigger antibody-dependent cytotoxicity (ADCC), as well as the production of pro-inflammatory cytokines and chemokines, such as IFN-γ, TNFα, IL-6, GM-CSF, and CCL5 (15, 16). In particular, NK-derived IFN-γ stands as a well-recognized key immunoregulatory factor in the shaping of anti-tumor adaptive immune responses, by modulating dendritic cells (DCs) and T-cell responses (17, 18). Moreover, the capability of CD16-initiated signals to regulate NK cell proliferation and death, under selective conditions, has been also shown (19, 20).

Human CD16 exhibits two extracellular Ig domains, a short cytoplasmic tail and a transmembrane domain that enables its association with ITAM-containing CD3ζ and FcεRIγ chains (21), which guarantee Syk- and ZAP-70-dependent signal transduction (16).

Multiple lines of evidence highlighted a functional superiority of memory compared with conventional NK cells, in response to stimulation through CD16, particularly in terms of cytokine production (6–8, 22). Indeed, memory NK cells exhibit a greatly enhanced ability to produce IFN-γ, as a consequence of hypo-methylated IFNG regulatory region (23), in response to activation via CD16, thus providing a prompt and powerful response against antibody-opsonized target cells.

The exploitation of memory NK cells in cancer combination immunotherapy may be highly attractive, because of their unique properties of CD16-dependent longevity and amplified functional responses. Indeed, CD16-triggered ADCC and phagocytosis, performed by NK cells and macrophages, respectively, are among the main immune-dependent mechanisms by which therapeutic monoclonal antibodies (mAbs) mediate tumor cell killing (24–27). Moreover, CD16-dependent immunomodulatory activity may contribute to the “vaccinal effect” of therapeutic tumor-targeting mAbs, i.e., the promotion of adaptive anti-tumor immune responses that confer long-term protection (17, 18, 28, 29). This concept is supported by the evidence that a single nucleotide polymorphism of the FCGR3A gene (c.559G>T, p.Phe158Val), encoding for a lower (FcγRIIIA-158F) or a higher (FcγRIIIA-158V) affinity allele of CD16 receptor, affects the clinical response to rituximab anti-CD20 mAb treatment that stands as a well-established first-line therapeutic option in several B cell malignancies (30, 31). More recently, new mAbs with enhanced affinity for CD16 have been generated. Among them, obinutuzumab, recently approved for clinical use (32–34), is a type II glycoengineered anti-CD20 mAb with an afucosylated crystallizable fragment (Fc) domain that binds to a CD20 epitope in a different space orientation and with a wider elbow-hinge angle with respect to the reference molecule rituximab (35).

Our recent data highlighted that distinct CD16 aggregation conditions, obtained through sustained contact with target cells opsonized by different anti-CD20 mAbs, differently promote the shift of NK cell functional program (36, 37).

Here, we address the capability of anti-CD20 mAbs to affect memory NK cell dynamics. We demonstrate that the co-culture with anti-CD20 mAb-opsonized targets selectively supports the *in vitro* expansion of *in vivo* primed memory NK cells, which phenotypically and functionally mirror their freshly isolated counterpart. CD16 engagement under quantitatively different affinity ligation conditions qualitatively impacts on memory NK-cell responses, being rituximab more efficient in supporting expansion, and obinutuzumab more active in inducing functional activation.

We also investigate the possible impact of CD16 functional allelic variants on memory NK cell *in vivo* and *in vitro* expansion.

## Materials and Methods

### Cell Systems and Anti-CD20 mAbs

Peripheral blood mononuclear cells (PBMCs) were freshly isolated from peripheral blood samples of anonymized healthy donors of Transfusion Center of Sapienza University of Rome, over a Ficoll-Hypaque (Cedarlane) density gradient. Written informed consent was obtained from blood donors, and both the informed consent form and procedure were approved by the Ethics Committee of Sapienza University of Rome. The study was conducted in accordance with the Declaration of Helsinki. The following human cell lines were used as targets: Raji CD20^+^ lymphoblastoid, provided by Dr. F. D. Batista (Cancer Research UK, London), and K562 erithroleukemia, obtained from ATCC. All cell lines were kept in culture for less than 2 consecutive months in 10% fetal calf serum (FCS)- and 1% l-glutamine (both from Euroclone)-containing RPMI 1640 and regularly checked for mycoplasma presence. The following anti-CD20 mAbs were used: the chimeric IgG1κ type I rituximab, the humanized IgG1κ type II glycoengineered obinutuzumab (GA101), and its nonglycoengineered parental molecule (GA101-WT), all kindly provided by Dr. Christian Klein, Roche Innovation Center Zurich (Schlieren, Switzerland).

### Immunostaining and Cytofluorometric Analysis

#### Phenotypic Characterization of NK Cells

Peripheral blood-derived and *in vitro* cultured mononuclear cell populations were identified by a combination of physical parameters and immunostaining with saturating concentrations of the following fluorochrome-conjugated mAbs: anti-CD3 PerCP (clone: SK7, cat #: 347344; BD Biosciences) or PerCP-Vio700 (clone: REA613, cat #: 130-109-465; Miltenyi Biotec), anti-CD56 APC (clone: B159, cat #: 555518; BD Biosciences) or APC-Vio770 (clone: REA 196, cat #: 130-100-694; Miltenyi Biotec), CD16 PE (clone: B73.1, cat #: 347617; BD Biosciences) or PE-Vio770 (clone: REA423, cat #: 130-106-706; Miltenyi Biotec), anti-FcεRIγ subunit FITC (polyclonal antibody, cat #: FCABS400F; Merck), anti-NKp46 APC (clone: REA808, cat #: 130-112-122; Miltenyi Biotec), anti-NKG2C PE (clone: 134591, cat #: FAB138P; R&D Systems), anti-PLZF PE (clone: Mags.21F7, cat #: 12-9320-82; ThermoFisher Scientific), anti-PD-1 PE (clone: EH12.2H7, cat #: 329906; BioLegend), and anti-PD-L1 APC (clone: 29E.2A3, cat #: 329708; BioLegend). Samples were stained for surface antigens for 30 min at 4°C, washed with PBS (Euroclone) containing 2% FCS and 2 mM EDTA (used for all washing steps), fixed with 2% paraformaldehyde for 20 min at room temperature (RT), washed, permeabilized with washing solution supplemented with 0.05% Triton-X 100 for 30 min at RT, and stained for intracellular antigens for 30 min at 4°C.

#### Characterization of FcγRIIIA-158V/F Allotype

The FcγRIIIA-158V/F allotype was determined on a cohort of 217 donors, as previously described (37). Briefly, PBMCs were stained with APC-conjugated anti-CD56 mAb and the following anti-CD16 mAbs: FITC-conjugated 3G8 (cat #: 555406; BD Biosciences) that binds to a not polymorphic epitope or FITC-conjugated MEM-154 (cat #: MAB-1457F; Immunological Science), whose binding to CD16 is dependent on the presence of valine (38). The ratio between the mean fluorescence intensity (MFI) of MEM-154 and 3G8 allows to classify the following three different phenotypes: F/F (ratio < 0.04), V/V (ratio > 0.62), and V/F (ratio between 0.15 and 0.48).

#### CD107a Mobilization and Intracellular IFN-γ Production

Freshly isolated PBMCs and their *in vitro* cultured counterparts were stimulated with Raji cells (2:1), opsonized or not with the minimum saturating dose of rituximab (1 μg/1 × 10^6^) or obinutuzumab (0.1 μg/1 × 10^6^), or with K562 targets, for 6 h at 37°C in the presence of PE-conjugated anti-CD107a mAb (clone: H4A3, cat #: 555801; BD Biosciences) and 50 µM Monensin (Golgi-stop; cat #: M5273; Merck). After the first hour, 10 µg/ml Brefeldin A (cat #: B7651; Merck) was added. At the end of stimulation, cells were washed with 5 mM EDTA containing PBS and then stained as earlier, adding the anti-IFN-γ APC (clone: B27, cat #: 554702; BD Biosciences) after permeabilization.

Samples were analyzed with a FACSCanto II (BD), and data were obtained with FlowJo vX.0.7 (TreeStar) software. Where required, twin samples were added and stained with isotype control mAb, used to set the threshold for antigen positivity. The values of cell counts and cytofluorimetric analysis percentages were used to obtain the absolute number of different NK cell subsets.

### *In Vitro* NK Cell Culture

Freshly isolated PBMC from healthy donors were seeded in round-bottomed 96-well plates (50,000 cells/well) and cultured for 10 days in RPMI 1640 medium supplemented with 10% FCS, 1% l-glutamine, 1% penicillin/streptomycin, and 100 U/ml of human recombinant IL-2 (cat #: AF-200-02; Peprotech); after 2 days, irradiated Raji cells (3000 rad), incubated or not with an excess of rituximab, obinutuzumab, or wt-obinutuzumab (GA101-WT) (all from Roche) for 20 min at RT, were washed and added to the cultures (25,000 cells/well). Recombinant hIL-2-containing medium (100 IU/ml) was half-replaced every 2 days. At day 10, cells were harvested and used for phenotypic and functional analyses. For functional assays, 10 ng/ml of IL-15 (cat #: AF-200-15; Peprotech) was added at day 7.

### HCMV Serostatus Analysis

Anti-HCMV plasma IgG titers were determined by CMV IgG Immulite 2000 System (Siemens Healthineers), according to the manufacturer’s instructions.

### Statistical Analysis

Differences between two groups were determined by two-tailed Mann–Whitney *U* test or Wilcoxon signed rank test, as appropriate. Analyses were performed using Prism v.6 (GraphPad Software) and SPSS v24.0 (IBM Italia SpA) software packages. Differences were considered to be statistically significant when *p* value was <0.05 (two sided).

## Results

### *In Vivo* Characterization of Memory NK Cell Compartment: Impact of FCGR3A-V158F Polymorphism

**Figure 1:**
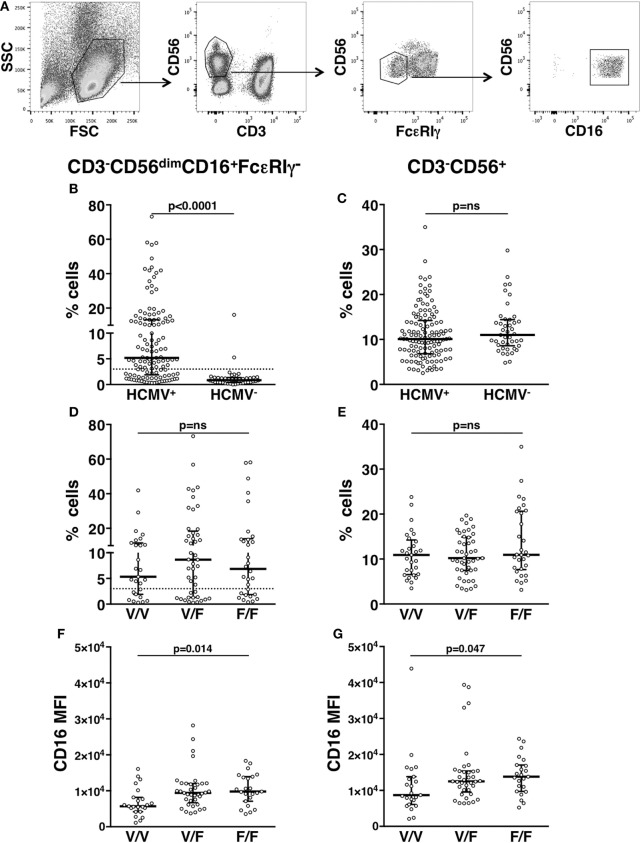
*In vivo* characterization of human memory natural killer (NK) cells. **(A)** Gating strategy for the identification of CD3^−^CD56^dim^CD16^+^FcεRIγ^−^ memory NK cells. **(B)** Percentage of memory NK cells (with respect to total NK cells) in the peripheral blood of human cytomegalovirus (HCMV) seropositive (HCMV^+^) and seronegative (HCMV^−^) healthy donors; dotted line marks the value of 3%. **(C)** Percentage of total CD3^−^CD56^+^ NK cells among peripheral blood mononuclear leukocytes, in the same individuals. **(D)** Percentage of memory NK cells (with respect to total NK cells) in the peripheral blood of HCMV^+^ healthy donors, stratified according to CD16 allotype; dotted line marks the value of 3%. **(E)** Percentage of total CD3^−^CD56^+^ NK cells among peripheral blood mononuclear leukocytes, in the same individuals. CD16 mean fluorescence intensity (MFI) on memory **(F)** and total **(G)** NK cells of HCMV^+^ healthy donors, stratified according to CD16 allotype. Bars represent median and 10–90 percentiles. *p* values of pairwise comparisons are from Mann–Whitney non-parametric test.

Our analysis of an extended cohort of 275 healthy donors evidenced that a discrete memory NK cell population, identified as CD3^-^CD56^dim^CD16^+^FcεRIγ^−^ (Figure 1A), and accounting for more than 3% of total CD3^-^CD56^+^ NK cells, was present in 52.4% of individuals (data not shown). In line with the literature (8–10), the presence of memory NK cells strictly correlated with HCMV seropositivity, as shown in serotyped individuals (Figure 1B). On the contrary, the percentage of total NK cells in HCMV-seropositive and -seronegative individuals was not significantly different (Figure 1C). FcεRIγ^−^ cells expressed other markers previously associated to memory NK cell profile (6, 8, 9): the percentage of NKG2C^+^ cells was higher whereas the fraction of NKp46^+^ cells was lower, with respect to the FcεRIγ^+^ counterpart. Moreover, PLZF transcription factor and CD16 expression levels were significantly lower in memory with respect to conventional NK cells (Figures S1A–D in Supplementary Material). Additionally, memory NK cells expressed PD-L1 at significantly lower levels than their conventional counterpart (Figure S1E in Supplementary Material).

It has been proposed that *in vivo* maintenance of a long-lasting memory NK cell population relies on CD16-dependent interaction with antiviral Ab-opsonized infected cells (7, 9). In order to analyze the impact of CD16 affinity for IgG on the *in vivo* development of memory NK cell compartment, HCMV^+^ individuals were stratified on the basis of CD16 allotype: V/V (homozygous for high-affinity allele, 29 donors), F/F (homozygous for low-affinity allele, 29 donors), and V/F (45 donors). The percentage of total NK cells and that of memory NK population were not significantly different among individuals bearing different CD16 allotypes (Figures 1D,E), although V/V donors displayed a tendentially reduced frequency of memory NK cells (Figure 1D). Notably, both memory (Figure 1F) and total (Figure 1G) NK cells of V/V donors expressed lower levels of CD16 receptor, with respect to F/F donors. Nevertheless, the reduced CD16 levels of memory NK cells with respect to conventional NK cells (Figure S1A in Supplementary Material) were maintained across the three CD16 allotypes (Figure S2 in Supplementary Material).

### Anti-CD20-Mediated CD16 Ligation Induces the Selective *In Vitro* Expansion of Memory NK Cells: Impact of HCMV Seropositivity Status and CD16 Affinity Ligation Conditions

**Figure 2:**
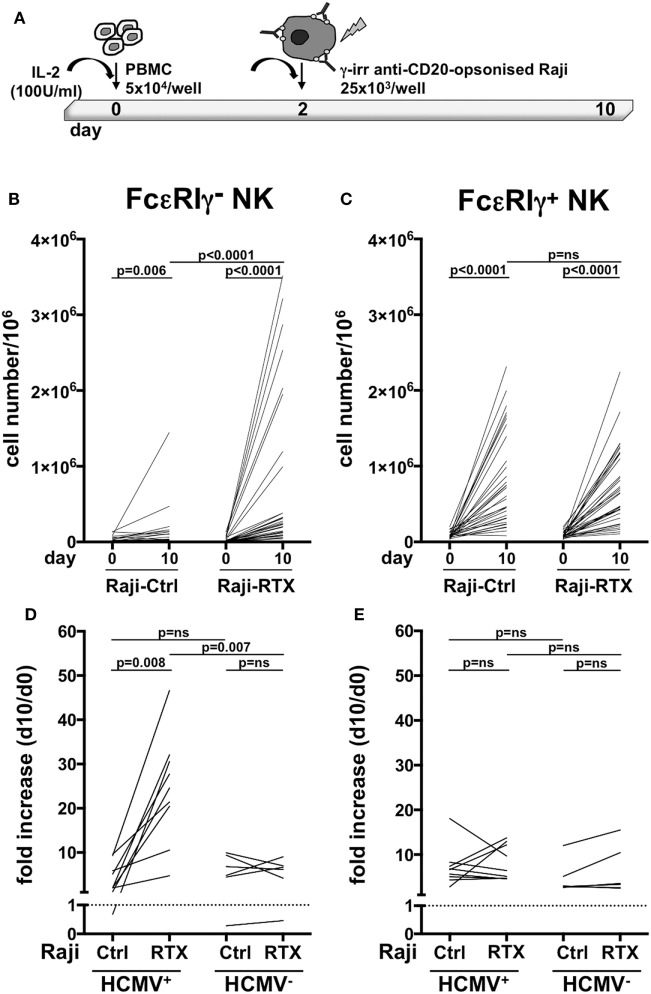
*In vitro* memory natural killer (NK) cell expansion upon interaction with rituximab-opsonized Raji lymphoblastoid cells. **(A)** Experimental setting of NK cell co-culture protocol. **(B)** Memory (FcεRIγ^−^) and **(C)** conventional (FcεRIγ^+^) CD3^−^CD56^dim^CD16^+^ NK cell numbers (per million mononuclear cells), at the start (day 0) and at the end (day 10) of co-culture with Raji cells, opsonized (RTX) or not (Ctrl) with rituximab anti-CD20 monoclonal antibody (mAb). **(D)** Memory (FcεRIγ^−^) and **(E)** conventional (FcεRIγ^+^) NK cell fold increase from individuals with less than 3% of memory NK cells. Donors were stratified according to human cytomegalovirus (HCMV) seropositivity status. Dotted line marks the value of 1. *p* values of pairwise comparisons are from Wilcoxon and Mann–Whitney non-parametric tests, as appropriate.

Previous work has demonstrated that *in vitro* expansion of memory NK cells critically requires Ab-opsonized virus-infected cells (7, 9). In order to assess whether therapeutic tumor-targeting mAbs may drive memory NK cells expansion, we co-cultured healthy donor PBMC that displayed a sizeable population of memory (>3% of NK cells), with Raji lymphoblastoid B cell line, opsonized or not with rituximab anti-CD20 mAb, in the presence of IL-2 (Figure 2A). While conventional NK cells efficiently underwent expansion (up to 50-fold, Figure 2C), independently from rituximab, memory NK cells proliferated much more markedly in the presence of rituximab (4.6–76.3-fold) than in its absence (0.1–16.7-fold) (Figure 2B), indicating an antibody requirement to support memory NK cell expansion.

In order to assess whether these *in vitro* culture conditions could promote memory NK cell generation, or rather amplify a pre-existing memory NK cell pool driven by *in vivo* HCMV exposure, we assayed memory NK cell expansion dynamics in donors that lacked a sizeable memory population (less than 3% of total NK cell population), obtained from either HCMV-seropositive and -seronegative individuals. Memory NK cells more vigorously proliferated in the presence of rituximab than in its absence, when derived from HCMV^+^ donors. At variance, rituximab addition did not appreciably affect *in vitro* expansion of memory NK cells from HCMV-seronegative individuals (Figure 2D). No significant differences in the extent of conventional NK cell subset proliferation were observed between HCMV^+^ and HCMV^−^ samples, either in the presence or in the absence of rituximab (Figure 2E).

Collectively, these data indicate that memory NK cells selectively expand *in vitro* in a rituximab-dependent manner and that such response critically requires a previous HCMV *in vivo* priming.

**Figure 3:**
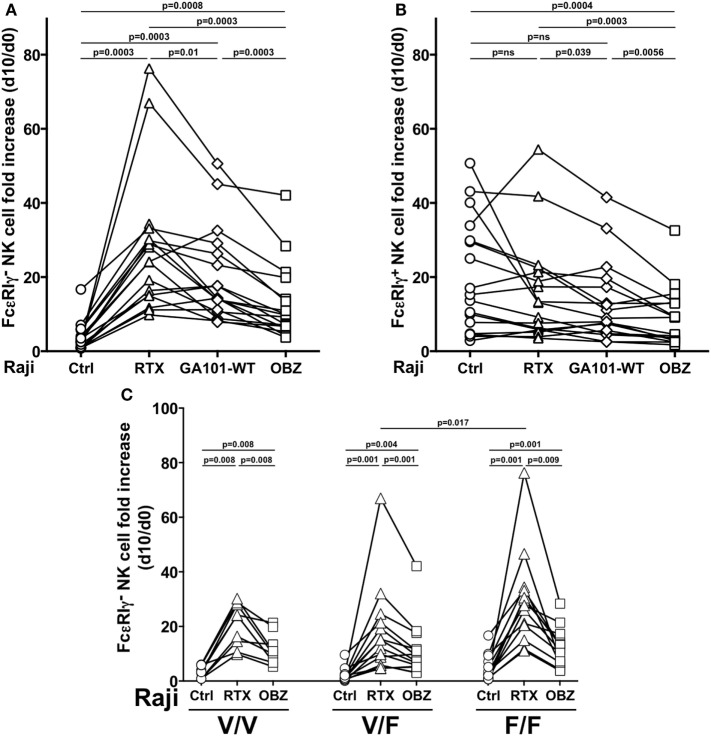
Effect of CD16 affinity ligation conditions on memory natural killer (NK) cell *in vitro* expansion from HCMV^+^ individuals. **(A)** Memory (FcεRIγ^−^) and **(B)** conventional (FcεRIγ^+^) CD3^−^CD56^dim^CD16^+^ NK cell fold increase, in response to the presence of Raji opsonized, or not (Ctrl), with rituximab (RTX), glycoengineered obinutuzumab (OBZ), or wild-type OBZ (GA101-WT). **(C)** Memory NK cell fold increase in response to rituximab or glycoengineered OBZ in samples stratified according to donor’s CD16 allotype; Only statistically significant comparisons are depicted. Donors reported in Figures 2B–C are included. *p* values of pairwise comparisons are from Wilcoxon and from Mann–Whitney non-parametric tests, as appropriate.

We then compared three anti-CD20 mAbs, endowed with low [rituximab or obinutuzumab wild-type, fucosylated parental mAb (GA101-WT)] or high (obinutuzumab) binding affinity for CD16, for their ability to drive memory NK cell proliferation; in this system, we could dissect the impact of FcR binding affinity from that of targeted epitope and binding orientation on CD20 molecule. Rituximab was markedly more efficient in inducing the expansion of memory NK cells, as compared with obinutuzumab; Fc glycoengineering only partially accounted for such difference, since the expansion capability of GA101-WT was intermediate, suggesting that factors other than CD16 affinity may impact on memory NK cell expansion (Figure 3A). Moreover, as conventional NK cell proliferation was negatively affected by obinutuzumab, it is possible that signals arising under high CD16 affinity ligation conditions may interfere with NK cell expansion (Figure 3B). However, T-cell proliferation was not negatively affected by obinutuzumab presence, in the same samples (data not shown).

Memory NK cells expressing all CD16 allotypes expanded in response to rituximab, with higher levels in low-affinity F/F, with respect to V/F individuals. Furthermore, obinutuzumab resulted less efficient in inducing memory NK cell proliferation independently from CD16 allotype, confirming its ability to overcome the affinity ligation differences due to CD16 polymorphism (Figure 3C).

### Phenotypic and Functional Profile of *In Vitro* Expanded Memory NK Cells

**Figure 4:**
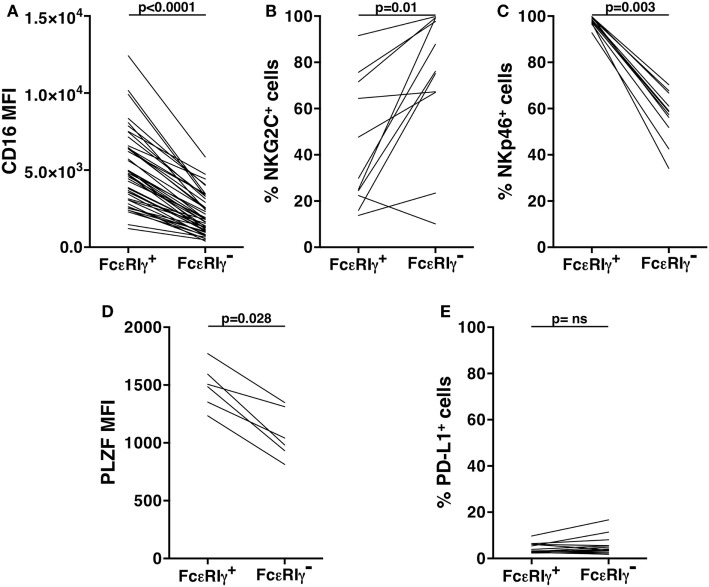
Phenotypic characterization of *in vitro* cultured human memory natural killer (NK) cells. CD16 mean fluorescence intensity (MFI) **(A)**, frequency of NKG2C^+^ **(B)**, and of NKp46^+^ **(C)** cells, pro-myelocytic leukemia zinc finger (PLZF) MFI **(D)**, and percentage of PD-L1^+^ **(E)** cells, were evaluated on memory (FcεRIγ^−^) and conventional (FcεRIγ^+^) CD3^−^CD56^dim^CD16^+^ NK cells, upon co-culture with rituximab-opsonized Raji cells. *p* values of pairwise comparisons are from Wilcoxon non-parametric test.

Consistent with what observed in the freshly isolated population (Figure S1 in Supplementary Material), *in vitro* cultured memory NK cells displayed a lower percentage of NKp46^+^ cells, as compared to FcεRIγ^+^ conventional population; the frequency of NKG2C^+^ memory NK cells remained significantly higher with respect to conventional ones; and similar to what observed in freshly isolated samples, the expression levels of PLZF transcription factor and CD16 were lower in cultured memory NK cells, as compared to conventional NK cells (Figures 4A–D). However, it must be noted that the expression of all these markers underwent marked modulation upon *in vitro* culture, on both memory and conventional NK cell subsets (compared with the respective panels of Figure S1 in Supplementary Material). Interestingly, PD-L1 expression stably remained on a low percentage of memory NK cells, while significantly declined on conventional NK cells, upon *in vitro* expansion (Figure 4E; Figure 1E in Supplementary Material).

The ability to mediate enhanced functional responses to Ab stimulation, particularly in terms of IFN-γ production, is a well-established characteristic of memory NK cells (6, 7, 9, 22).

**Figure 5:**
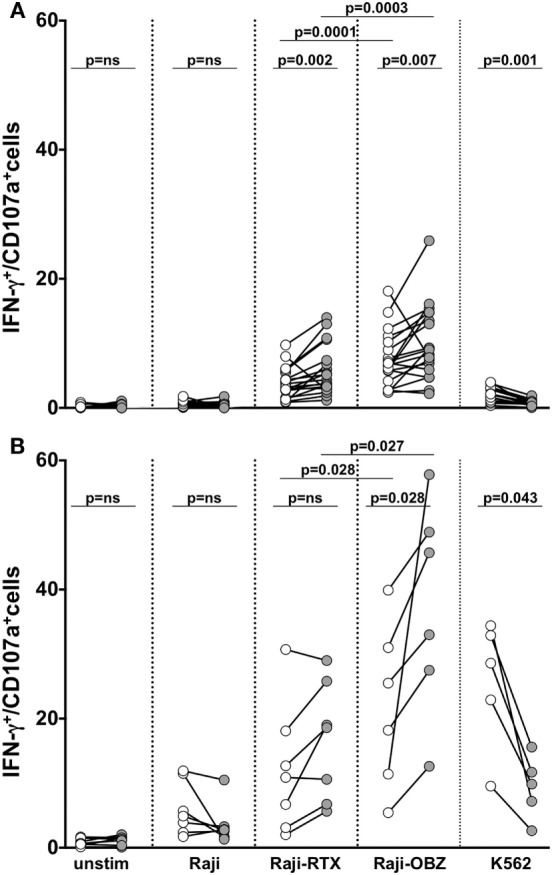
Functional responsiveness of fresh and *in vitro* cultured memory natural killer (NK) cells. Fresh **(A)** and cultured **(B)** memory (filled symbols) and conventional (empty symbols) NK cells were stimulated as indicated, and the percentage of CD107a/^+^IFN-γ^+^ was evaluated by immunostaining and FACS analysis. *p* values of pairwise comparisons are from Wilcoxon non-parametric test.

We assessed memory NK cell responsiveness to CD16 or to direct target stimulation. In freshly isolated populations, upon CD16 stimulation obtained with rituximab or obinutuzumab-opsonized targets, the frequency of memory NK cells able to mediate both degranulation and IFN-γ production was significantly higher with respect to the conventional counterpart. Notably, obinutuzumab-mediated stimulation resulted more efficient in the induction of multifunctional responses in both memory and conventional NK cells, with respect to rituximab stimulation (Figure 5A).

Basal responsiveness of *in vitro* expanded NK cells was enhanced, as a consequence of the exposure to IL-2 plus IL-15 during the last 3 days of culture. In these conditions, obinutuzumab-mediated stimulation was able to induce a significantly higher response rate in memory with respect to conventional NK cells (Figure 5B). Differently, memory NK cell responses to rituximab-coated target cells were only tendentially higher than conventional NK cells, possibly in dependence on the markedly low CD16 expression level (see Figure 4A). Furthermore, in line with the literature and similarly to what observed on their fresh counterparts, mAb-expanded cultured memory NK cells displayed a defective ability to respond to K562 target cells (6).

## Discussion

Natural killer cell’s role in tumor immunosurveillance, as well as their involvement in immune-dependent therapeutic efficacy of tumor-targeting mAbs, is widely recognized (27, 39). NK cell functional and phenotypic heterogeneity, together with the recent identification of a long-lived and hyper-responsive subset, named adaptive or memory, have raised much interest, to the end of a better exploitation of this anti-tumor effector in the development of innovative combination immunotherapeutic strategies.

The present work provides a further characterization of freshly isolated human memory NK cells, identified as CD3^−^CD56^dim^CD16^+^ FcεRIγ^−^, and defines the ability of therapeutic mAb-coated tumor cells to efficiently drive their *in vitro* expansion from HCMV-seropositive healthy donors’ PBMC. Indeed, we developed an effective *in vitro* culture system, consisting of a 10-day co-culture of PBMC with irradiated lymphoblastoid Raji cells opsonized with anti-CD20 mAbs, in IL-2-containing medium. Our results demonstrate the strict dependence of memory NK cell proliferation on the presence of opsonizing mAb, as their expansion in the presence of not opsonized targets was only marginal. These data strongly support the concept that CD16-initiated signals are crucially involved in triggering the proliferation of this specific subset. In accordance, previous evidence had shown the capacity of anti-viral IgG-opsonized cells to induce memory NK cells proliferation (8, 9). Differently, conventional NK cells were capable of effectively and comparably expand upon co-culture with either not opsonized or therapeutic mAb-coated feeder cells, thus suggesting that cell–cell interactions with surface ligands expressed by Raji lymphoma cells, together with cytokines, provide sufficient proliferative signals to them. Indeed, the capability of EBV^+^ lymphoblastoid cell lines to induce NK cell proliferation has been widely reported (40), and the crucial role of 2B4/CD48 interaction in this context has been recently proposed (41). Moreover, CD16-dependent NK cell proliferation has been recently reported, in a 21-day culture protocol under selected co-stimulation conditions, but no evaluation of the memory NK cell compartment was performed (20). It is conceivable that CD16-dependent memory NK cell proliferation also relies on multiple accessory signals, conveyed by cell–cell contacts and soluble mediators; in this context, the synergistic effect of antisera and virus-infected cells was reported (8, 9). In our system, ligands expressed by Raji lymphoblastoid B cells may provide accessory proliferative signals to memory NK cells; among them, CD2 ligand, CD58, has been shown to co-stimulate memory NK cell responses (22). Moreover, monocyte-derived IL-12, probably stimulated through FcγR engagement by anti-CD20-opsonized targets, likely mediates a critical contribution through the up-regulation of CD25, as demonstrated by a recent report (12).

Our results identify donor HCMV-seropositive status as a prerequisite to allow anti-CD20 mAb-dependent memory NK cell *in vitro* expansion. In fact, *in vitro* memory NK cell proliferation could be obtained from HCMV^+^, but not from HMCV^−^ donors that *in vivo* lacked a sizeable (>3% of total NK cells) memory NK cell population. These data suggest that HCMV exposure is indispensable for the priming of a memory NK cell pool and are in accordance with multiple lines of evidence that have demonstrated the unique ability of HCMV to epigenetically shape a NKG2C^+^ memory NK compartment, and the capability of viral reactivation to promote a long-lasting expansion of the memory NK cell pool (1–5). Furthermore, *in vitro* expansion of NKG2C^+^ memory NK cells could be achieved by co-culturing NK cells with HCMV-infected fibroblasts or HLA-E-expressing feeder (11, 12, 42). In these conditions, the interaction between the activating receptor CD94/NKG2C and its cellular ligand HLA-E, in combination with inflammatory cytokines, was critical for their expansion.

As a consequence of the lack of FcεRIγ chain, and in line with previous reports (6, 12), we found that freshly isolated memory NK cells displayed lower levels of FcεRIγ-coupled activating receptors, such as NKp46 and CD16, and of PLZF transcription factor, whose deficiency is responsible for the silencing of FcεRIγ gene (8), as compared to their conventional counterpart; moreover, as previously reported (4, 10, 11), NKG2C^+^ cells were more abundant within memory NK cell pool. Although the expression of these markers was dramatically modulated upon *in vitro* culture, their distinctive expression pattern on memory *vs* conventional NK cells was remarkably maintained. Interestingly, our results identify the lower expression of PD-L1, ligand of PD-1 immune checkpoint, as another distinctive feature of freshly isolated memory NK cells, as compared with conventional counterpart. In this regard, although the presence of PD1^+^ NK cells in a limited percentage of HCMV^+^ healthy individuals has been previously reported (43), the expression of PD-L1 ligand on human NK cells remains mostly unexplored.

Here, we have addressed the impact of CD16 affinity ligation on memory NK cell *in vitro* and *in vivo* expansion, and functional activation. We observed that CD16F158V polymorphism dictating receptor binding affinity for Ab Fc region (30, 31) does not affect the frequency of memory NK cells *in vivo*, in an extended cohort of CD16 genotyped individuals. Nevertheless, CD16 expression levels on either total NK cells or memory NK cell fraction were higher in low (F/F) with respect to high (V/V) affinity individuals, probably as a consequence of a reduced rate of Ab-induced CD16 internalization. Interestingly, *in vitro* memory NK cell expansion was heavily affected by CD16 ligation conditions promoted by anti-CD20 therapeutic mAbs. In fact, rituximab, a low-affinity ligand for CD16, much more efficiently drove memory NK cell expansion than obinutuzumab, whose glycoengineering allows a high-affinity interaction with the receptor. The lower capability of obinutuzumab to trigger memory NK cell proliferation was maintained across all CD16 allotypes, in accordance with its capacity to overcome polymorphism-dependent CD16 affinity differences (37, 44). Along this line, the rate of memory NK cell expansion in response to rituximab was superior in F/F low-affinity homozygous with respect to V/F donors, suggesting that their proliferation may benefit from a low-affinity CD16 ligation.

Proliferation of both memory and conventional NK cells was negatively affected by the presence of obinutuzumab, when compared with rituximab reference mAb. These data suggest that ligation conditions alter the balance between cell death and proliferation stimulated by CD16 cross-linking. Indeed, the capability of CD16-induced signals to induce apoptosis in NK cells has been earlier reported (19, 45). Such observation may offer an explanation for the protracted reduction of circulating NK cell levels observed in obinutuzumab-receiving patients (46). Moreover, these data imply that CD16-generated signaling pathways, although not necessary to drive their *in vitro* expansion, may also operate in conventional NK cells. Co-culture with non-glycoengineered obinutuzumab (GA101-WT)-opsonized targets only partially restored memory NK cell expansion ability, suggesting that other factors than higher affinity for CD16 are responsible for obinutuzumab reduced ability to support memory NK cell proliferation. Indeed, as a class II mAb, it is endowed with a reduced capacity to induce CD20 down-regulation on target cells and a higher capability to induce direct target cells death (47) that could impact stimulation efficiency. However, it is also possible that different CD16 affinity ligation conditions impact on memory NK cell expansion kinetics.

A key feature of memory NK cells is their superior Ab-dependent functional response, with respect to conventional ones (6–9, 22). Indeed, despite the lower CD16 expression, they have been shown to more efficiently mediate multifunctional responses, i.e., degranulation and IFN-γ production, upon stimulation via ADCC. This characteristic is confirmed by our data, as either freshly isolated or *in vitro* cultured memory NK cells displayed a higher frequency of multifunctional (IFN-γ^+^CD107a^+^) cells, upon triggering with anti-CD20-opsonized target cells. The apparent conflict between higher CD16-triggered functional responses and lower surface receptor levels may be explained by the exclusive coupling of CD16 to CD3ζ chain in memory NK cells that, thanks to ITAM motif quantitative differences (3 ITAM in CD3ζ *vs* 1 ITAM in FcεRIγ), may lead to more robust and efficient biochemical signals (15). Moreover, the residual levels of CD3ζ chain may preserve the CD2/CD58 co-stimulatory interaction (48).

Interestingly, stimulation with obinutuzumab-coated targets promoted a more efficient activation of either fresh or *in vitro* expanded memory NK cells and led to enhanced degranulation and IFN-γ production, with respect to rituximab. The enhanced obinutuzumab-triggered response, as expected, was also observed in conventional NK cell populations. These data highlight a signal dichotomy downstream of CD16 engagement, being the high-affinity ligation an added value for functional activation and the low affinity useful for proliferative signals. The reduction of NKp46 levels may explain the reduced ability of fresh and *in vitro* cultured memory NK cell to mediate effector functions in response to stimulation with K562 target cell, being its recognition largely dependent on this receptor (49). However, NKG2C^+^ memory NK cells from HCMV-reactivating patients efficiently produced IFN-γ upon K562 stimulation (5, 50), indicating that the up-regulation of other activating receptors may compensate for the NKp46 defect.

In conclusion, we add new insights on the molecular factors governing the expansion and activation of memory NK cells. In a therapeutic perspective, they represent a particularly attractive tool of cancer immunotherapy, especially in mAb-based regimens where the enhanced responsiveness to mAb-coated targets could significantly impact therapeutic efficacy. These results may guide future attempts to combine the adoptive transfer of *ex vivo* expanded memory NK cells, or their *in vivo* manipulation, with therapeutic mAb administration.

Finally, it is worth investigating the possible contribution of this long-lived NK cell population to the recently described “vaccinal effect” of therapeutic mAbs. Indeed, thanks to their amplified capability to produce cytokines upon CD16 stimulation, memory NK cells could participate to the development of adaptive anti-tumor immune responses, required for the long-term protection of treated patients (27–29).

## Ethics Statement

Written informed consent was obtained from blood donors, and both the informed consent form and procedure were approved by the Ethics Committee of Sapienza University of Rome. The study was conducted in accordance with the Declaration of Helsinki.

## Author Contributions

RG and GP planned the study, analyzed data, and wrote the manuscript. CC and SB performed the experiments, analyzed the data, provided critical inputs, and contributed to writing of the manuscript. CP and LF performed the experiments, analyzed the data, and provided critical inputs. SM and OT provided experimental and critical expertises. AS provided critical inputs and contributed to discussion. All authors contributed to the editing of the article and gave approval.

## Conflict of Interest Statement

The submitted work was carried out in the absence of any personal, professional, or financial relationships that could potentially be construed as a conflict of interest.
