# Supplementary material for: Tumor-Targeting Anti-CD20 Antibodies Mediate In Vitro Expansion of Memory Natural Killer Cells: Impact of CD16 Affinity Ligation Conditions and In Vivo Priming
Source: Front Immunol. 2018 May 11;9:1031. doi: 10.3389/fimmu.2018.01031 (PMC5958227; doi:10.3389/fimmu.2018.01031)
Supplement: Supplementary file 1 [file image_1.PDF]

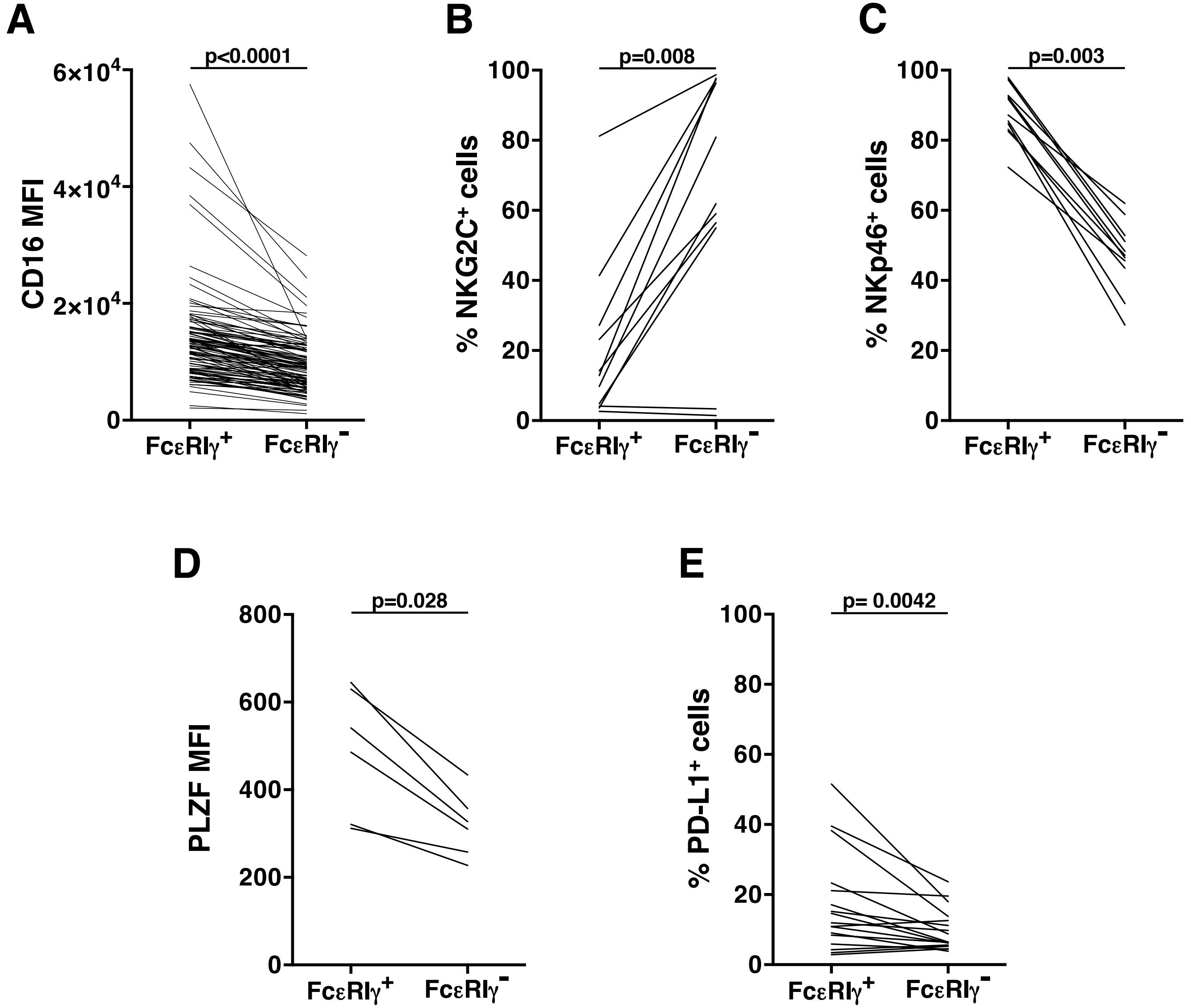

Supplementary Figure 1. Phenotypic characterization of fresh human memory NK cells. CD16 mean fluorescence intensity (MFI) (**A**), frequency of NKG2C$^+$ (**B**), and of NKp46$^+$ (**C**) cells, PLZF mean fluorescence intensity (MFI) (**D**), and percentage of PD-L1$^+$ (**E**) cells, were evaluated on memory (FcεRIγ$^-$) and conventional (FcεRIγ$^+$) CD3$^-$CD56^{dim}CD16$^+$ NK cells in the peripheral blood of HCMV$^+$ healthy donors (individuals shown in Figure 1 are included).
P values of pairwise comparisons are from Wilcoxon non-parametric test.

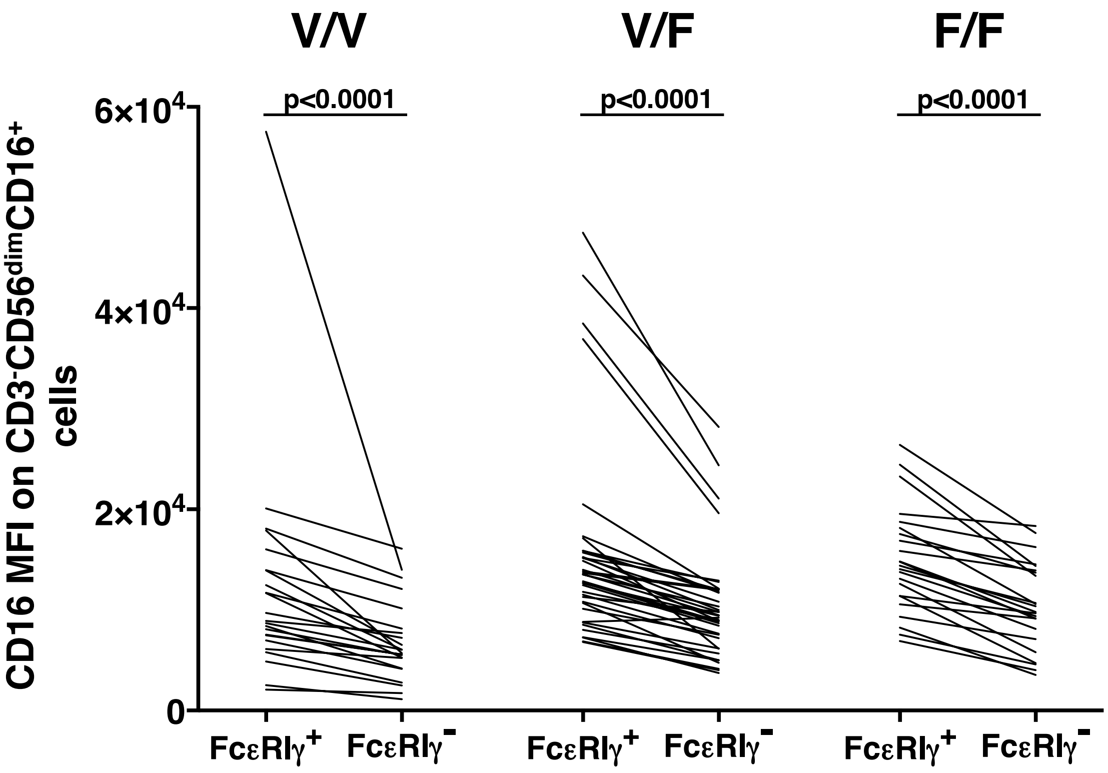

Supplementary Figure 2. CD16 receptor levels on NK cell subsets in individuals bearing different CD16 allotypes. CD16 mean fluorescence intensity (MFI) was evaluated on memory ($Fc\varepsilon RI\gamma^-$) and conventional ($Fc\varepsilon RI\gamma^+$) $CD3^-CD56^{dim}CD16^+$ NK cells in the peripheral blood of $HCMV^+$ healthy individuals, in samples stratified according to donor's CD16 allotype (individuals shown in Figure 1 are included).

P values of pairwise comparisons are from Wilcoxon non-parametric test.
